# Supplementary figures and images for: Telomere length sensitive regulation of interleukin receptor 1 type 1 (IL1R1) by the shelterin protein TRF2 modulates immune signalling in the tumour microenvironment
Source: eLife. 2024 Dec 27;13:RP95106. doi: 10.7554/eLife.95106 (PMC11677240; doi:10.7554/eLife.95106)

Figure 1- figure supplement 2 I

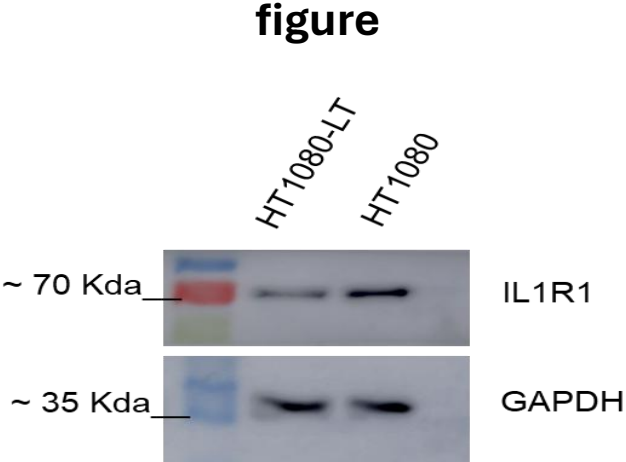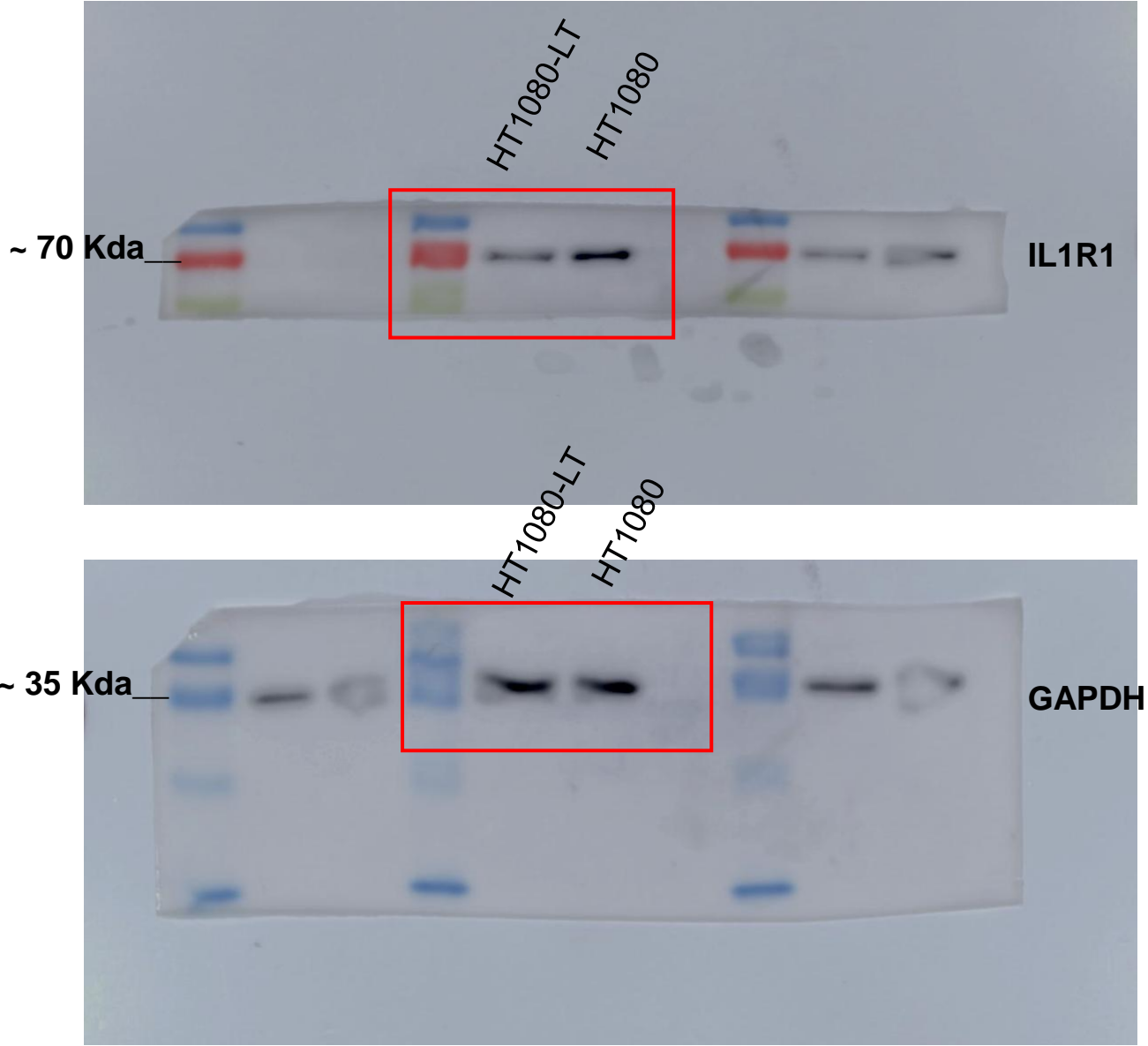

Supplement: Figure 1—figure supplement 2—source data 2. [file elife-95106-fig1-figsupp2-data2.zip › Figure 1_ figure supplement 2_source data 2_western blots fig I.pdf]

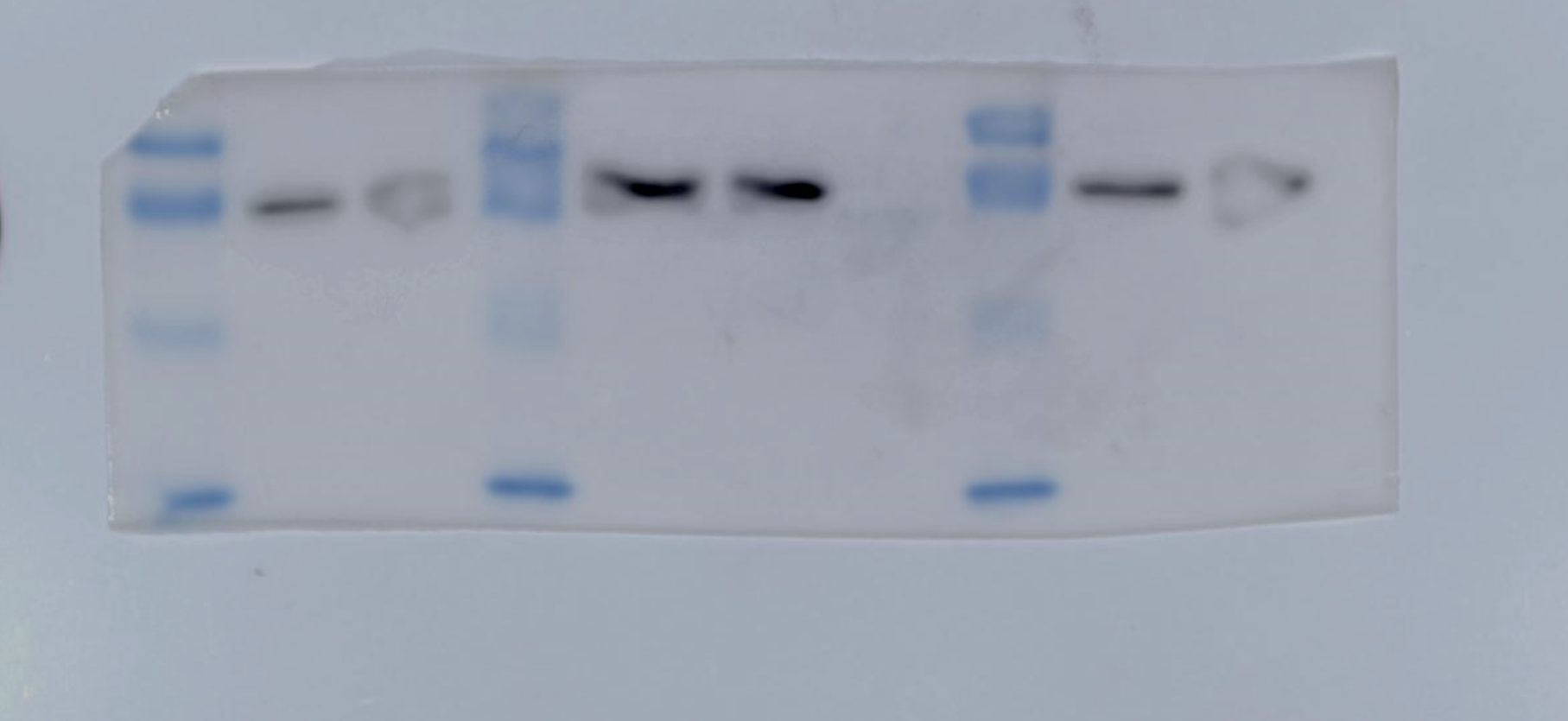

Supplement: Figure 1—figure supplement 2—source data 3. [file elife-95106-fig1-figsupp2-data3.zip › Figure 1_ figure supplement 2_source data 3_western blots fig I/Figure 1 figure supplement 2I GAPDH.tif]

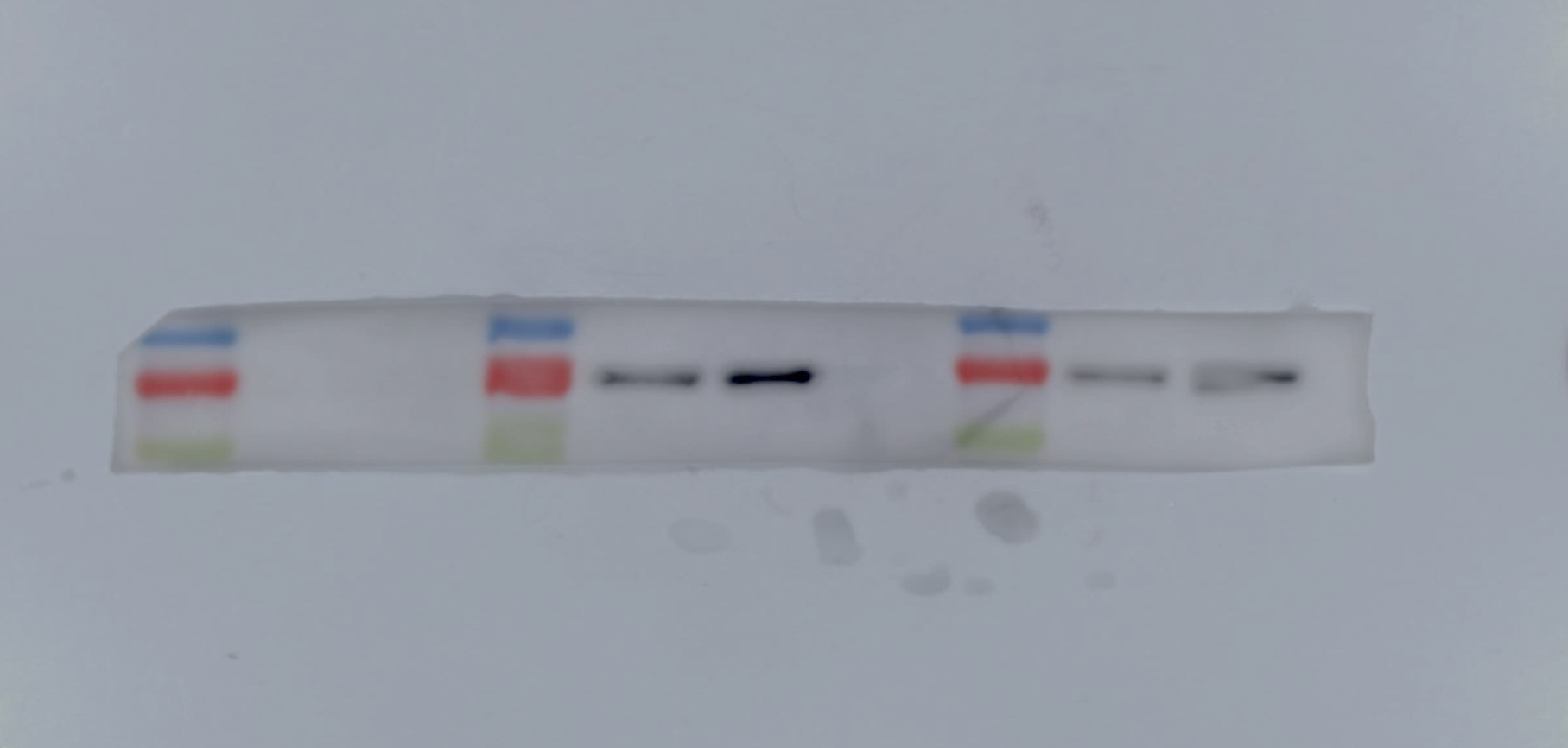

Supplement: Figure 1—figure supplement 2—source data 3. [file elife-95106-fig1-figsupp2-data3.zip › Figure 1_ figure supplement 2_source data 3_western blots fig I/Figure 1 figure supplement 2I IL1R1.tif]

**Figure 2D**

**figure**

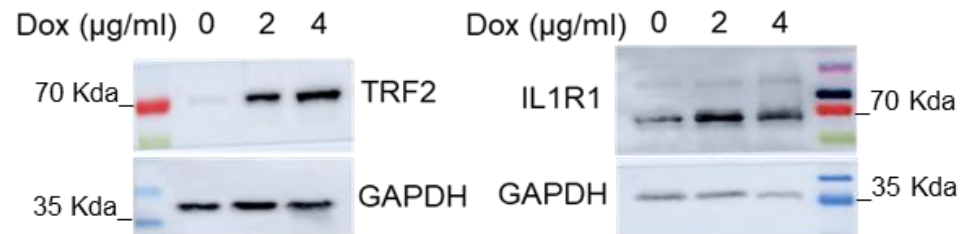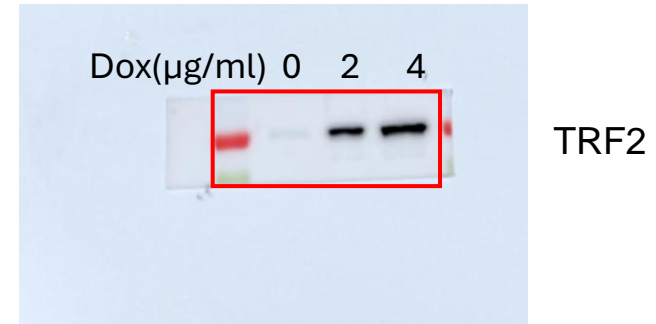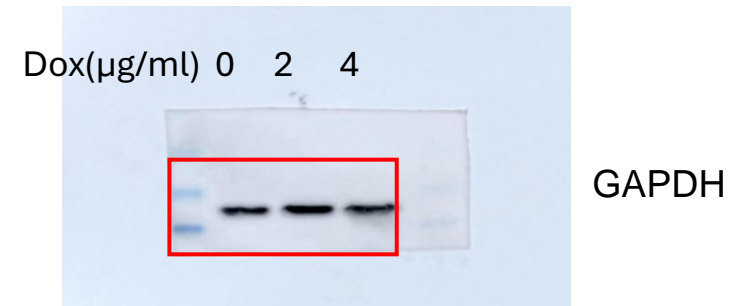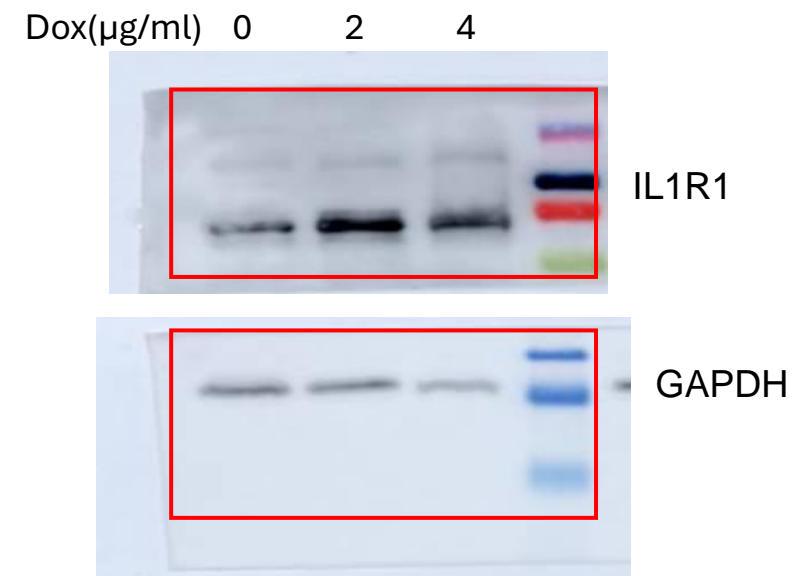

Supplement: Figure 2—source data 2. [file elife-95106-fig2-data2.zip › Figure 2_ source data 2_western blots 2D.pdf]

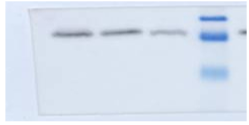

Supplement: Figure 2—source data 3. [file elife-95106-fig2-data3.zip › Figure 2_ source data 3_western blots 2D/Figure 2D GAPDH for IL1R1.tif]

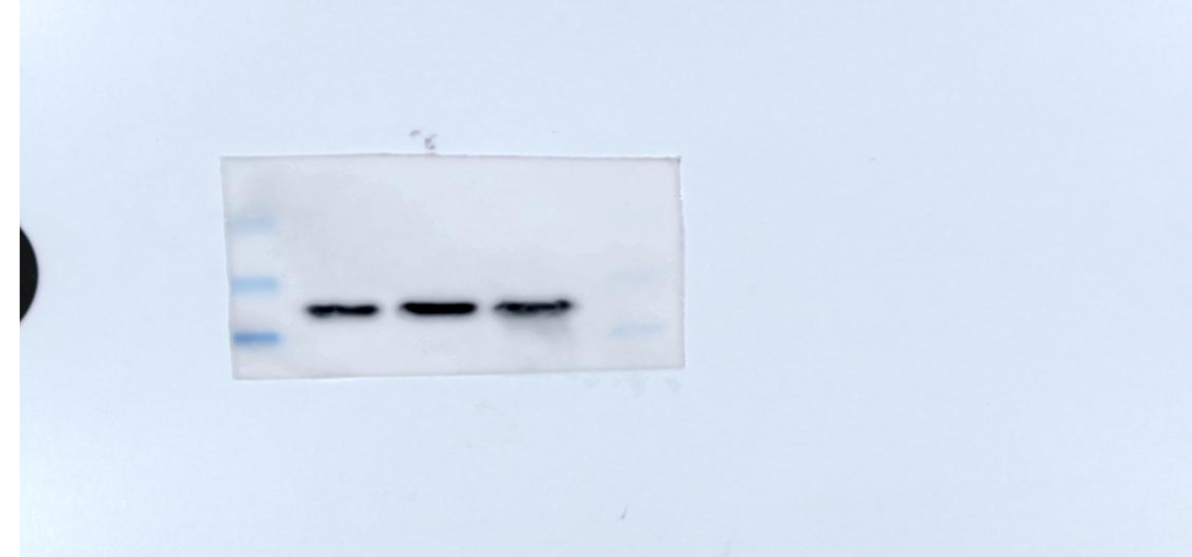

Supplement: Figure 2—source data 3. [file elife-95106-fig2-data3.zip › Figure 2_ source data 3_western blots 2D/Figure 2D GAPDH for TRF2.tif]

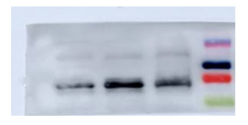

Supplement: Figure 2—source data 3. [file elife-95106-fig2-data3.zip › Figure 2_ source data 3_western blots 2D/Figure 2D IL1R1.tif]

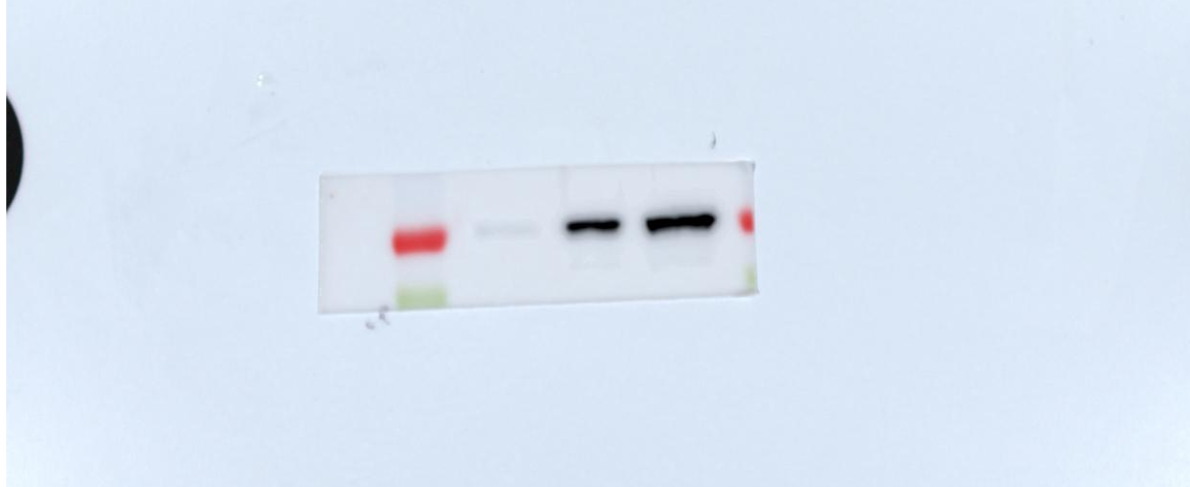

Supplement: Figure 2—source data 3. [file elife-95106-fig2-data3.zip › Figure 2_ source data 3_western blots 2D/Figure 2D TRF2.tif]

Figure 2 –figure supplement 1A

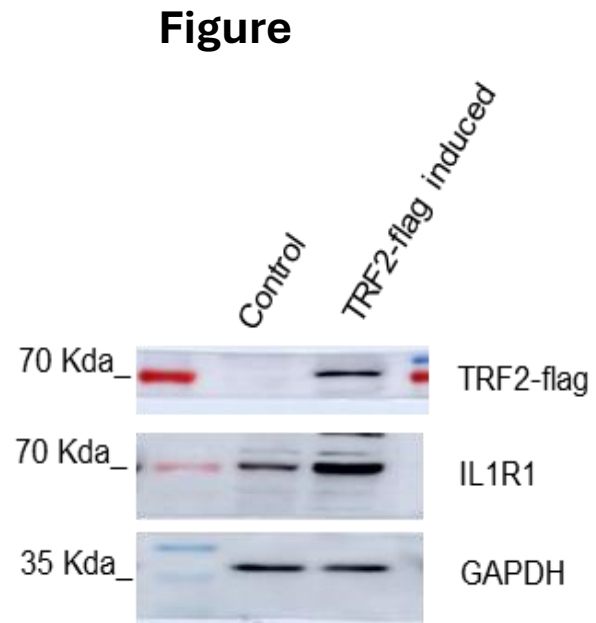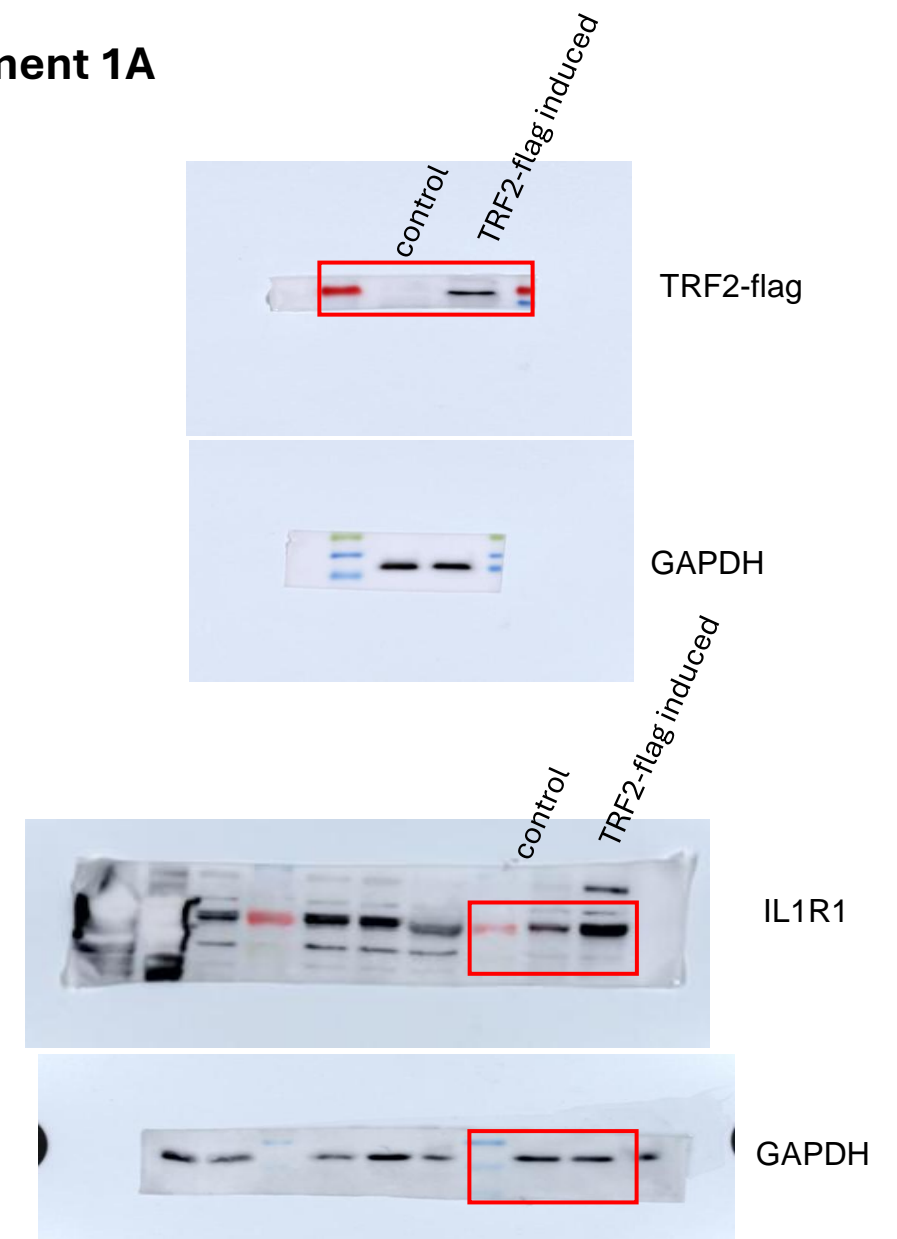

**Figure**

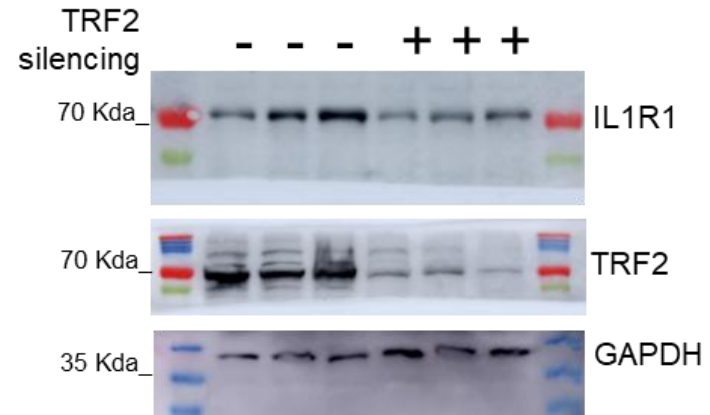

TRF2  
silencing

- - - + + +

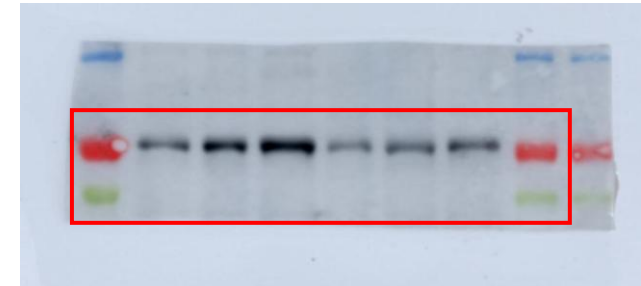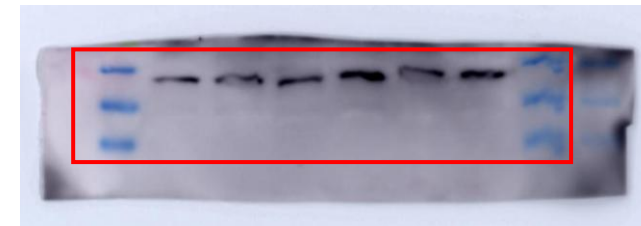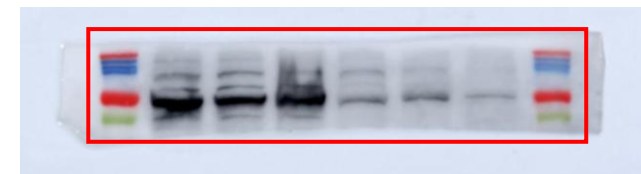

**Figure 2 –figure supplement 1 B**

**Figure**

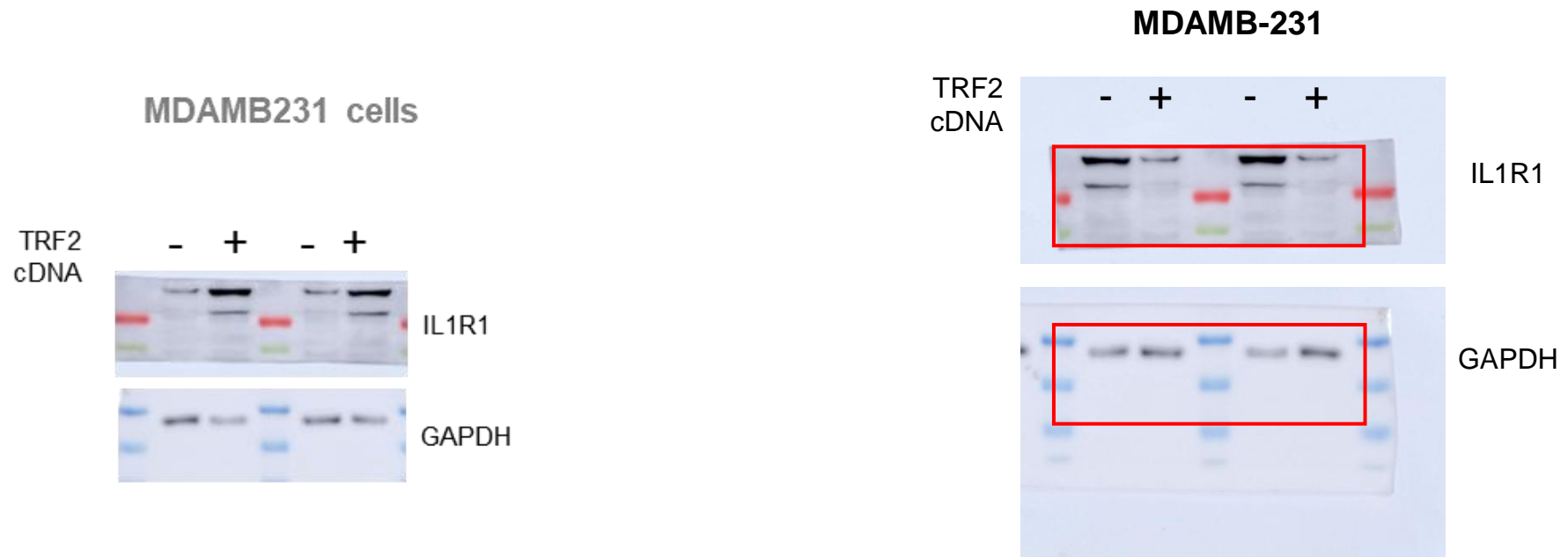

Supplement: Figure 2—figure supplement 1—source data 2. [file elife-95106-fig2-figsupp1-data2.zip › Figure 2_ figure supplement 1_source data 2_western blots fig A_B.pdf]

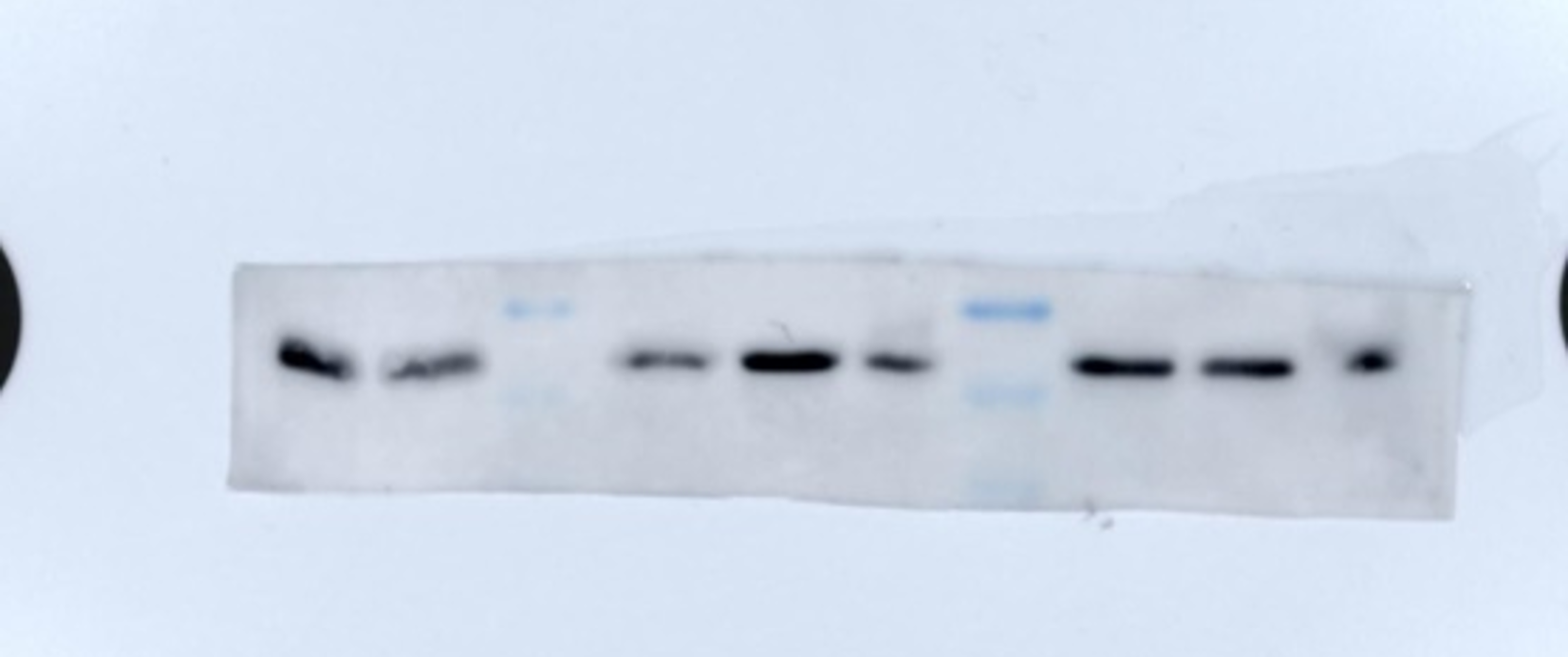

Supplement: Figure 2—figure supplement 1—source data 3. [file elife-95106-fig2-figsupp1-data3.zip › Figure 2_ figure supplement 1_source data 3_western blots fig A B/Figure 2 figure supplement 1 A left GAPDH for IL1R1.tif]

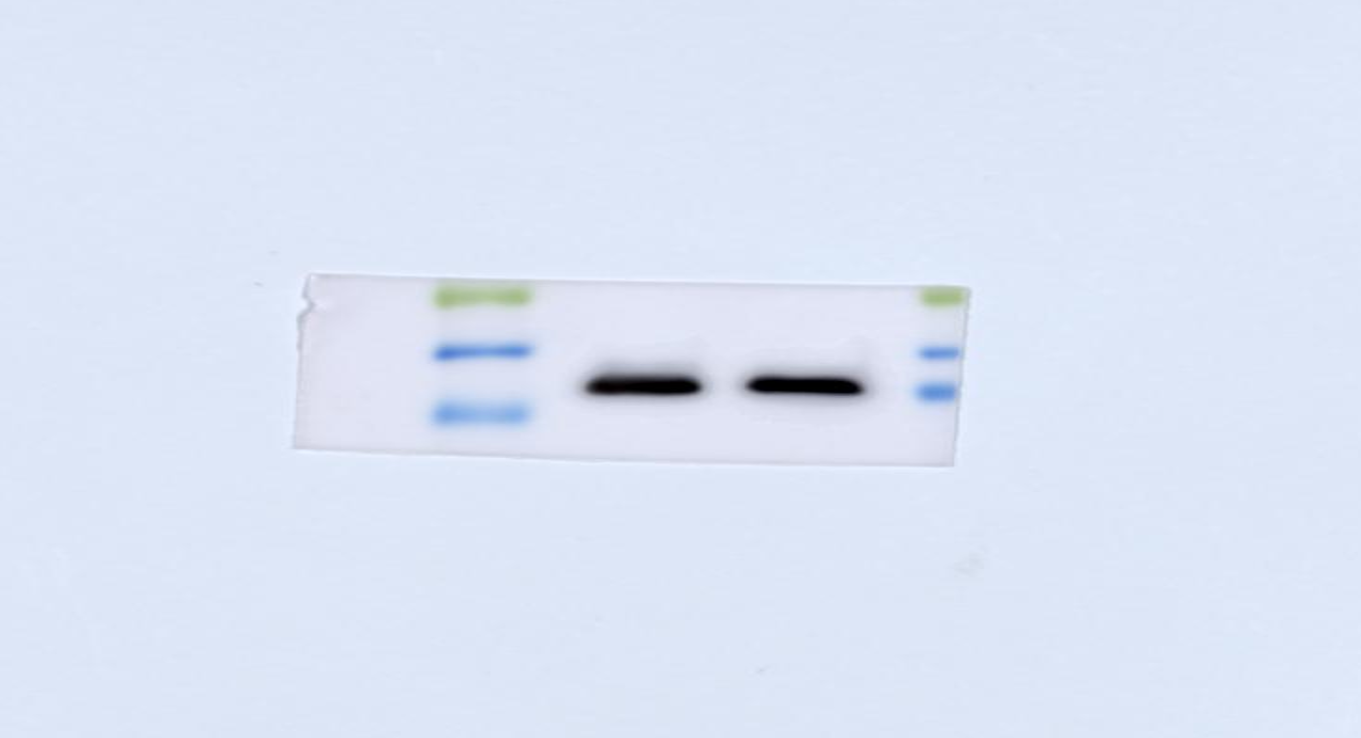

Supplement: Figure 2—figure supplement 1—source data 3. [file elife-95106-fig2-figsupp1-data3.zip › Figure 2_ figure supplement 1_source data 3_western blots fig A B/Figure 2 figure supplement 1 A left GAPDH for TRF2 flag.tif]

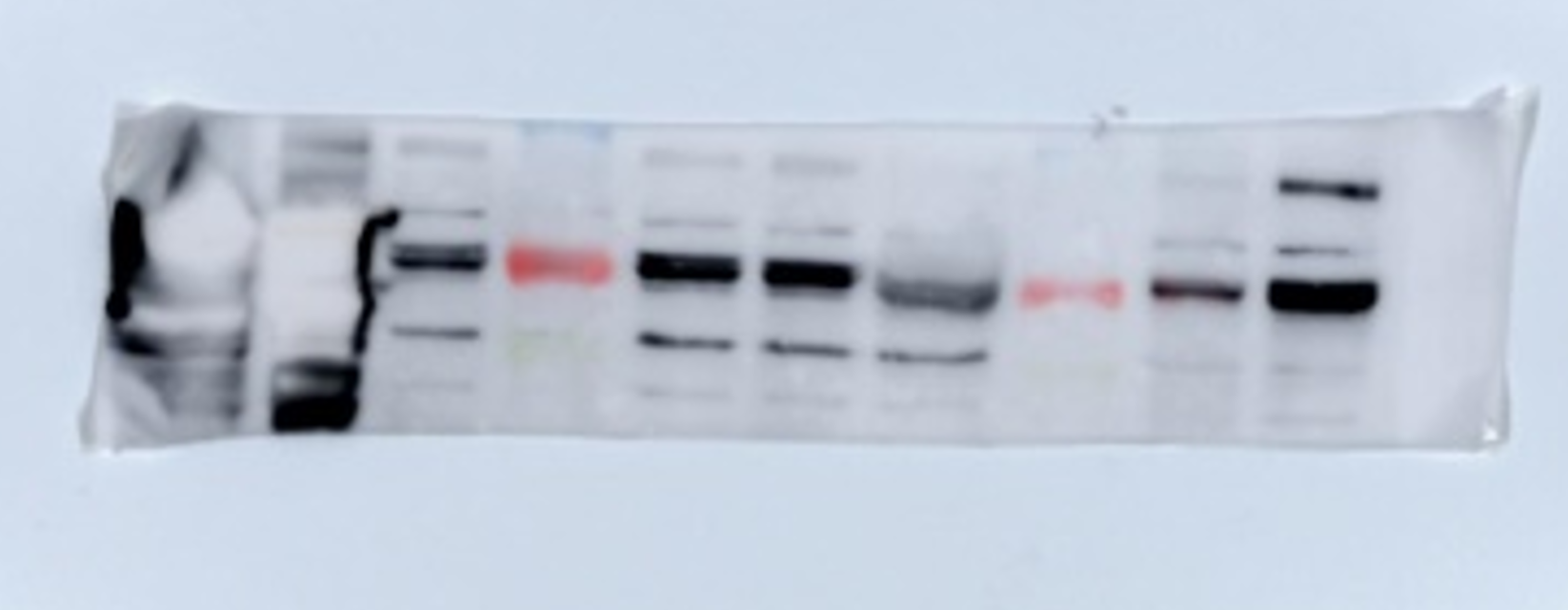

Supplement: Figure 2—figure supplement 1—source data 3. [file elife-95106-fig2-figsupp1-data3.zip › Figure 2_ figure supplement 1_source data 3_western blots fig A B/Figure 2 figure supplement 1 A left IL1R1.tif]

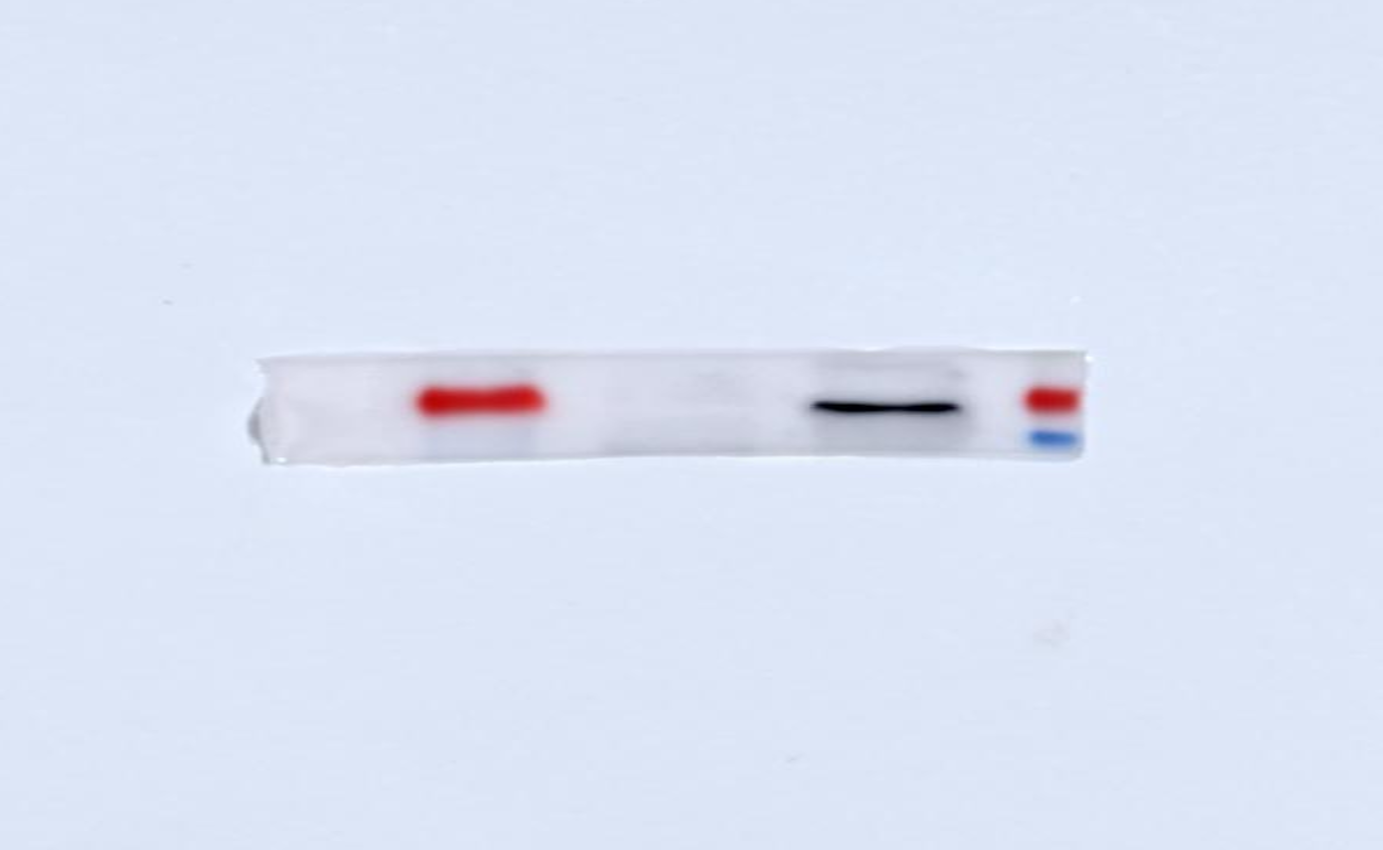

Supplement: Figure 2—figure supplement 1—source data 3. [file elife-95106-fig2-figsupp1-data3.zip › Figure 2_ figure supplement 1_source data 3_western blots fig A B/Figure 2 figure supplement 1 A left TRF2 flag.tif]

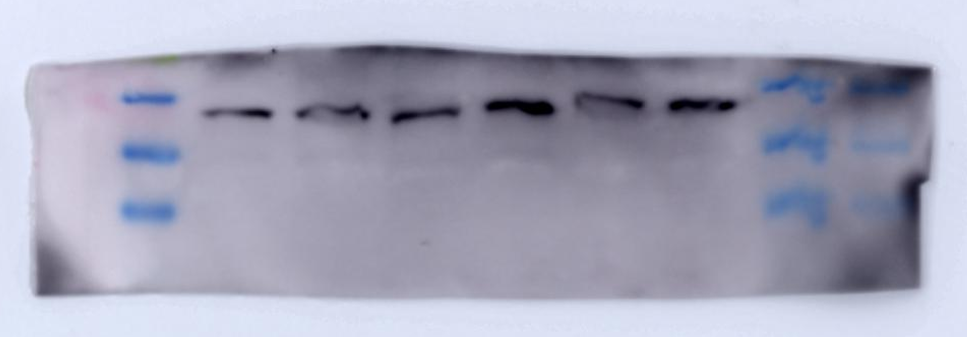

Supplement: Figure 2—figure supplement 1—source data 3. [file elife-95106-fig2-figsupp1-data3.zip › Figure 2_ figure supplement 1_source data 3_western blots fig A B/Figure 2 figure supplement 1 A right GAPDH for IL1R1.tif]

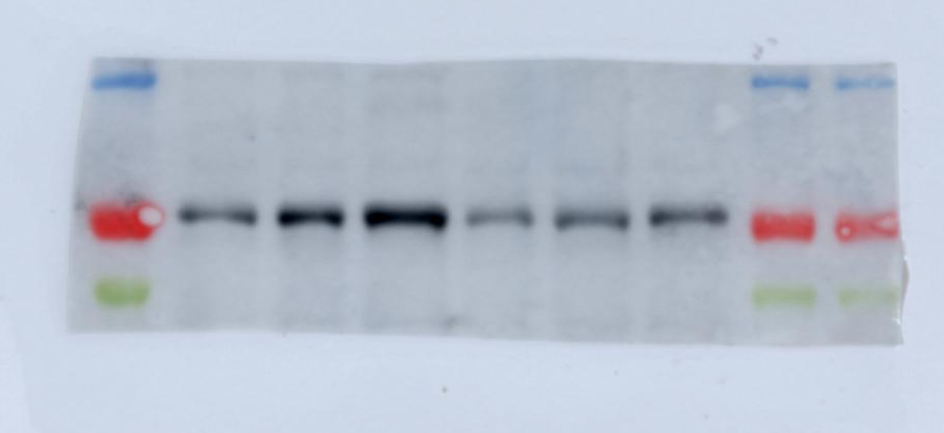

Supplement: Figure 2—figure supplement 1—source data 3. [file elife-95106-fig2-figsupp1-data3.zip › Figure 2_ figure supplement 1_source data 3_western blots fig A B/Figure 2 figure supplement 1 A right IL1R1.tif]

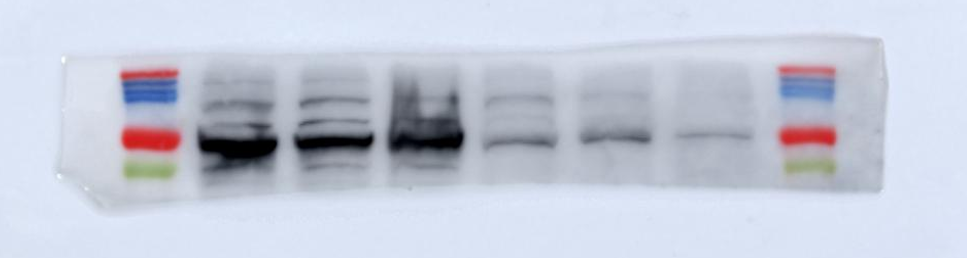

Supplement: Figure 2—figure supplement 1—source data 3. [file elife-95106-fig2-figsupp1-data3.zip › Figure 2_ figure supplement 1_source data 3_western blots fig A B/Figure 2 figure supplement 1 A right TRF2.tif]

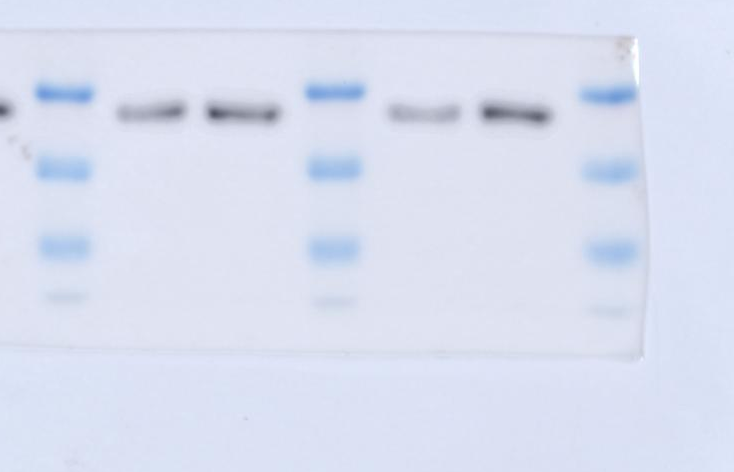

Supplement: Figure 2—figure supplement 1—source data 3. [file elife-95106-fig2-figsupp1-data3.zip › Figure 2_ figure supplement 1_source data 3_western blots fig A B/Figure 2 figure supplement 1 B GAPDH.tif]

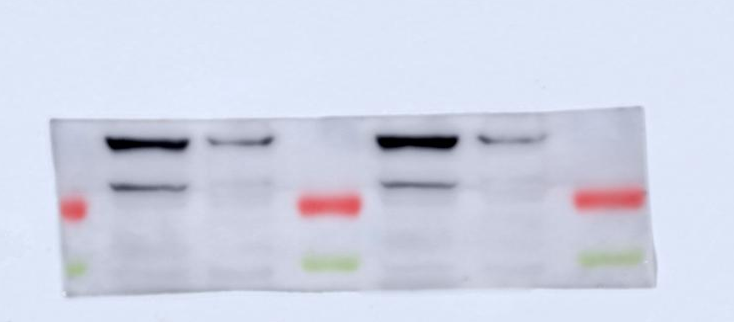

Supplement: Figure 2—figure supplement 1—source data 3. [file elife-95106-fig2-figsupp1-data3.zip › Figure 2_ figure supplement 1_source data 3_western blots fig A B/Figure 2 figure supplement 1 B IL1R1.tif]

Figure 3 E

Figure

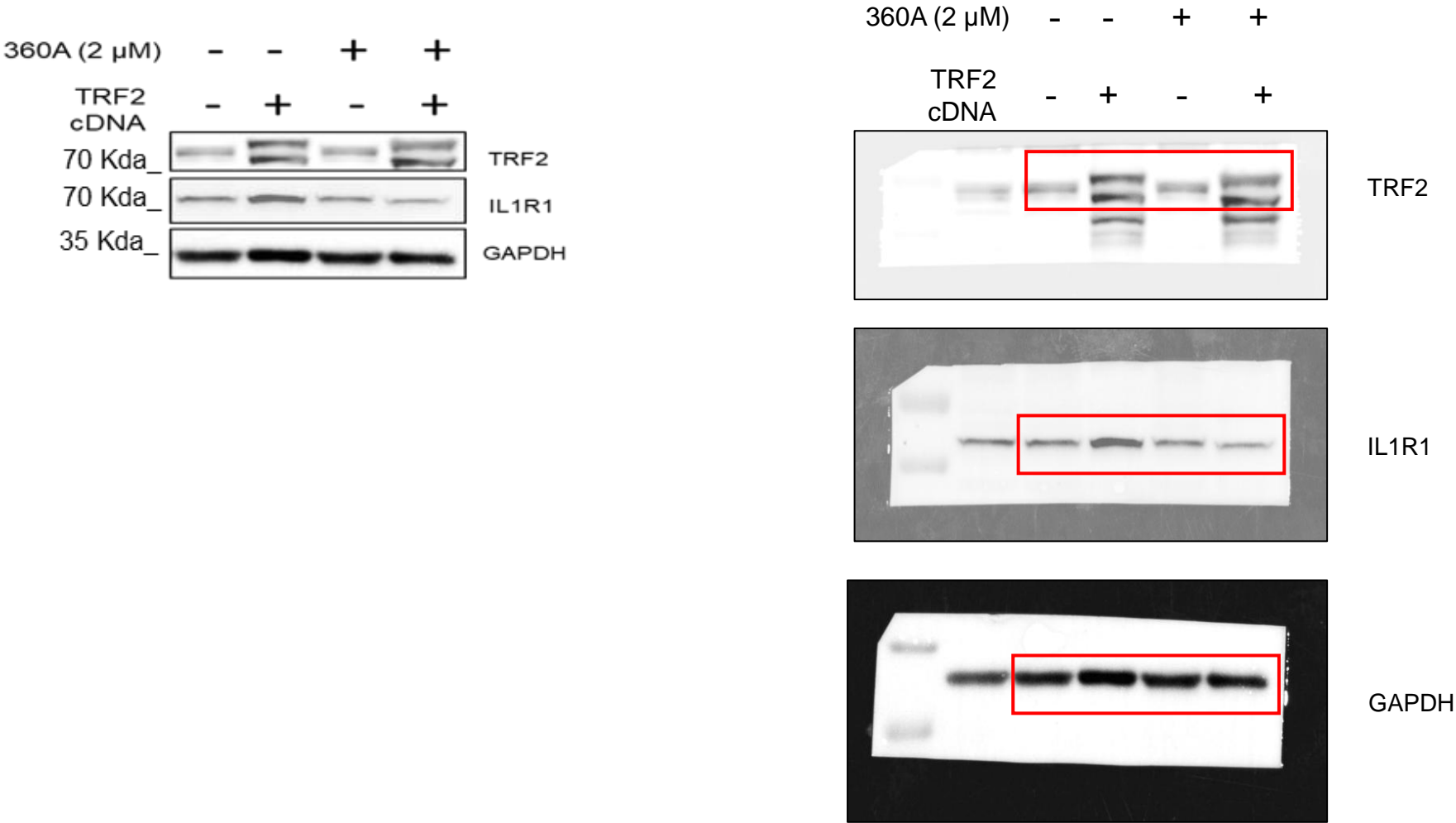

Supplement: Figure 3—source data 2. [file elife-95106-fig3-data2.zip › Figure 3_ source data 2_western blots 3E.pdf]

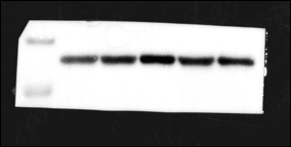

Supplement: Figure 3—source data 3. [file elife-95106-fig3-data3.zip › Figure 3_ source data 3_western blots 3E/Figure 3E GAPDH.tif]

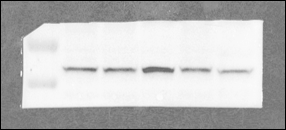

Supplement: Figure 3—source data 3. [file elife-95106-fig3-data3.zip › Figure 3_ source data 3_western blots 3E/Figure 3E IL1R1.tif]

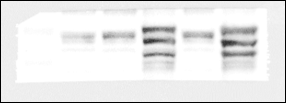

Supplement: Figure 3—source data 3. [file elife-95106-fig3-data3.zip › Figure 3_ source data 3_western blots 3E/Figure 3E TRF2.tif]

Figure 4-figure supplement 1C

Figure

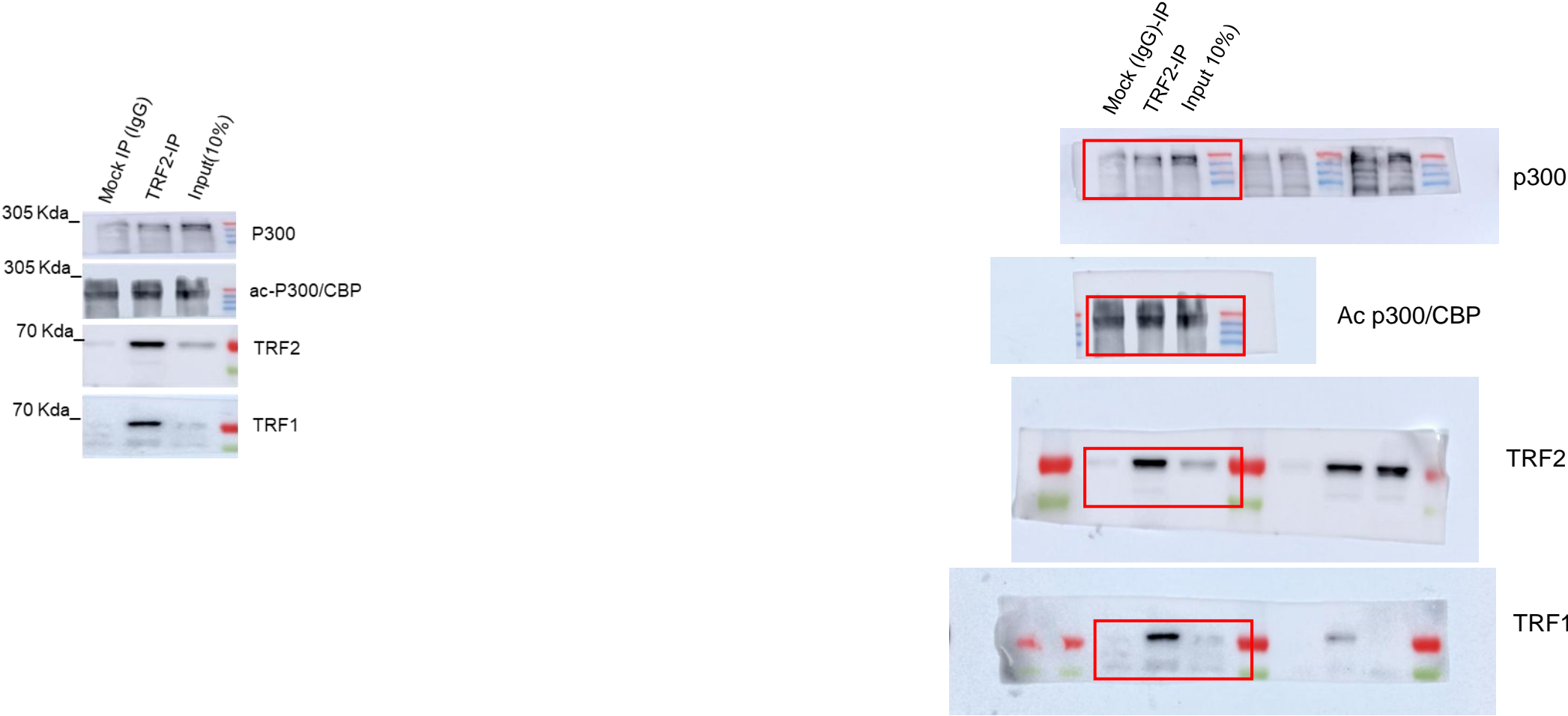

Supplement: Figure 4—figure supplement 1—source data 2. [file elife-95106-fig4-figsupp1-data2.zip › Figure 4_ figure supplement 1_source data 2_western blots fig C.pdf]

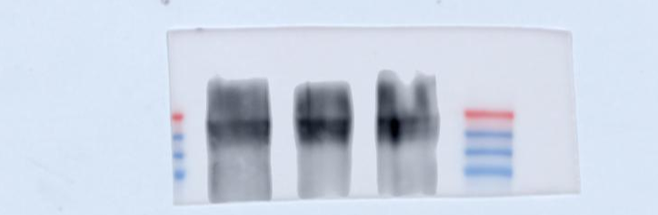

Supplement: Figure 4—figure supplement 1—source data 3. [file elife-95106-fig4-figsupp1-data3.zip › Figure 4_ figure supplement 1_source data 3_western blots fig C/Figure 4_figure supplement1C_IP acP300 CBP.tif]

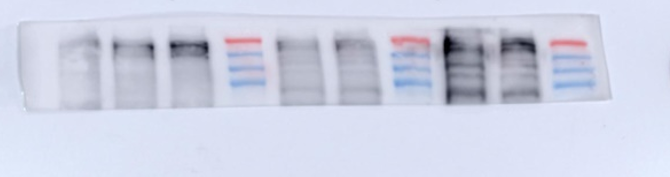

Supplement: Figure 4—figure supplement 1—source data 3. [file elife-95106-fig4-figsupp1-data3.zip › Figure 4_ figure supplement 1_source data 3_western blots fig C/Figure 4_figure supplement1C_IP P300.tif]

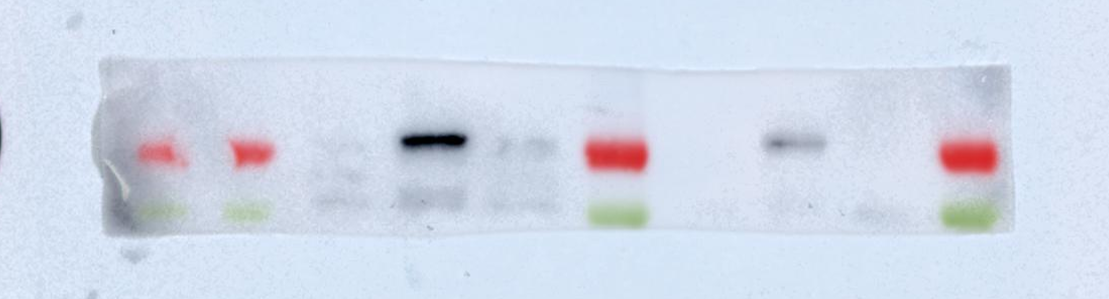

Supplement: Figure 4—figure supplement 1—source data 3. [file elife-95106-fig4-figsupp1-data3.zip › Figure 4_ figure supplement 1_source data 3_western blots fig C/Figure 4_figure supplement1C_IP TRF1.tif]

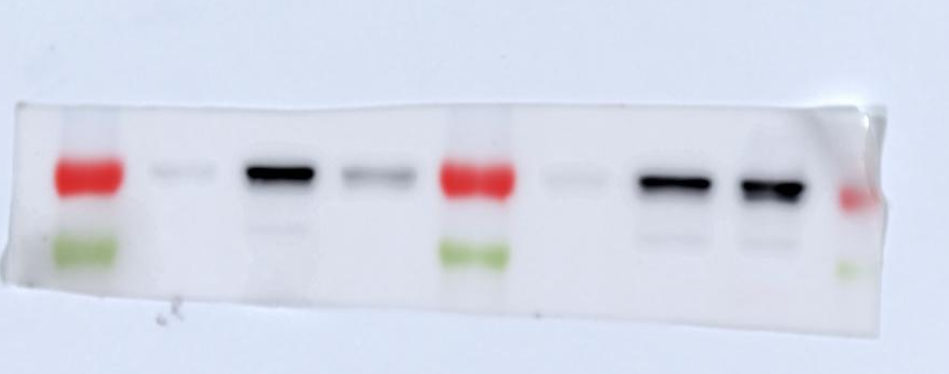

Supplement: Figure 4—figure supplement 1—source data 3. [file elife-95106-fig4-figsupp1-data3.zip › Figure 4_ figure supplement 1_source data 3_western blots fig C/Figure 4_figure supplement1C_IP TRF2.tif]

**Figure 5C**

**Figure**

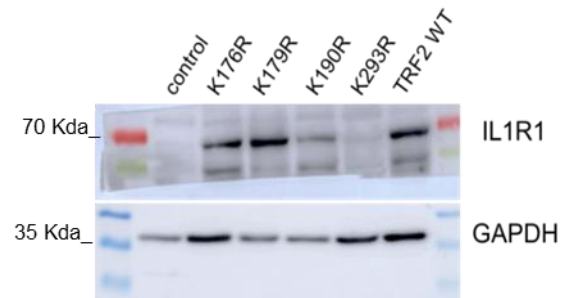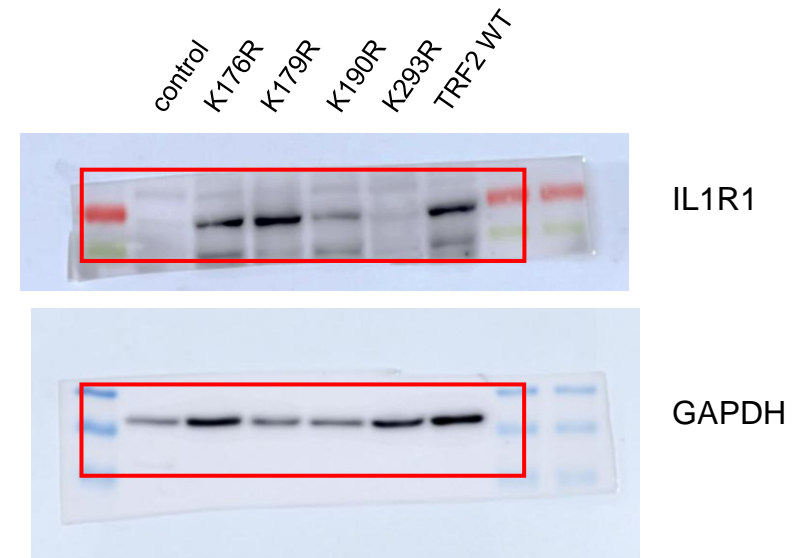

Supplement: Figure 5—source data 2. [file elife-95106-fig5-data2.zip › Figure 5_ source data 2_western blots fig C.pdf]

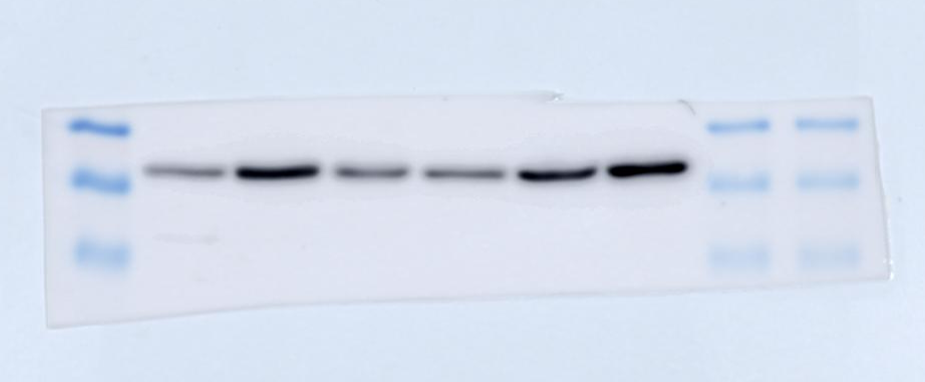

Supplement: Figure 5—source data 3. [file elife-95106-fig5-data3.zip › Figure 5_ source data 3_western blots 5C/Figure 5C GAPDH.tif]

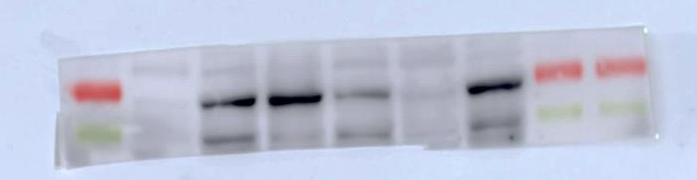

Supplement: Figure 5—source data 3. [file elife-95106-fig5-data3.zip › Figure 5_ source data 3_western blots 5C/Figure 5C IL1R1.tif]

Figure 5-figure supplement 1 A

Figure

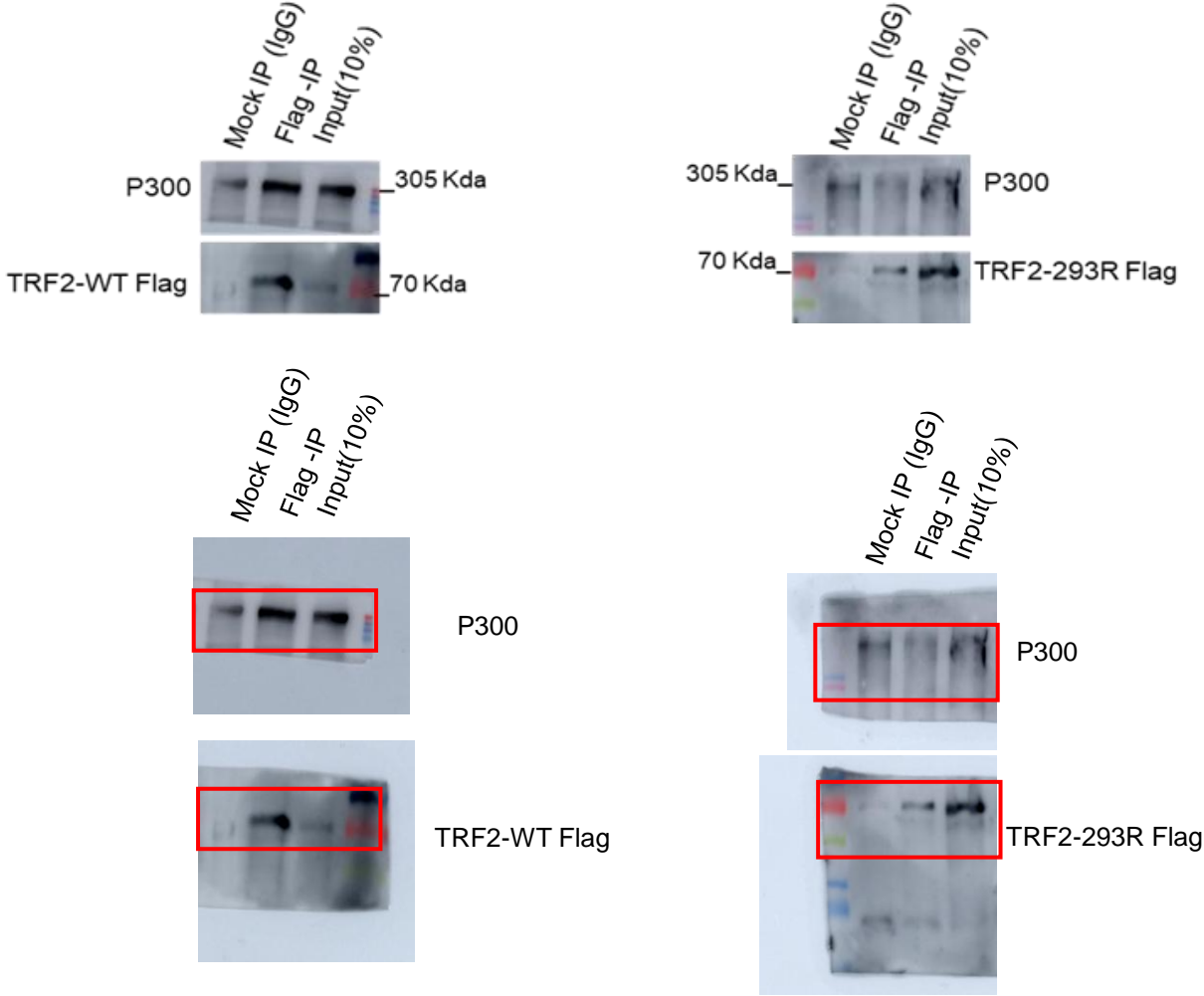

Supplement: Figure 5—figure supplement 1—source data 2. [file elife-95106-fig5-figsupp1-data2.zip › Figure 5_ figure supplement 1_source data 2_western blots fig A.pdf]

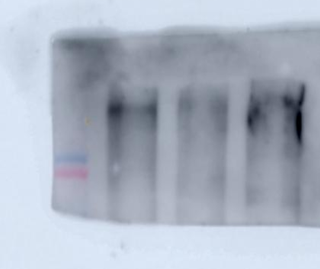

Supplement: Figure 5—figure supplement 1—source data 3. [file elife-95106-fig5-figsupp1-data3.zip › Figure 5_ figure supplement 1_source data 3_western blots fig A/Figure 5 figure supplement 1A p300 for TRF2 293R.tif]

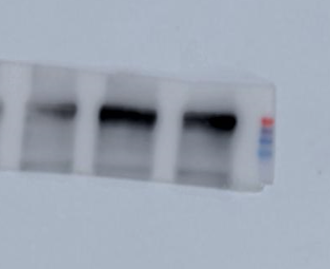

Supplement: Figure 5—figure supplement 1—source data 3. [file elife-95106-fig5-figsupp1-data3.zip › Figure 5_ figure supplement 1_source data 3_western blots fig A/Figure 5 figure supplement 1A p300 for TRF2 wt.tif]

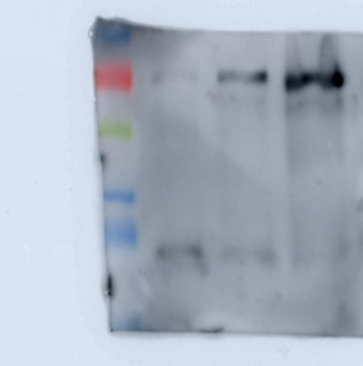

Supplement: Figure 5—figure supplement 1—source data 3. [file elife-95106-fig5-figsupp1-data3.zip › Figure 5_ figure supplement 1_source data 3_western blots fig A/Figure 5 figure supplement 1A TRF2 flag for TRF2 293R.tif]

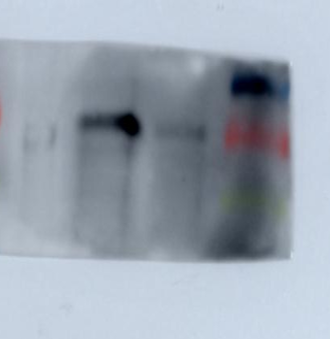

Supplement: Figure 5—figure supplement 1—source data 3. [file elife-95106-fig5-figsupp1-data3.zip › Figure 5_ figure supplement 1_source data 3_western blots fig A/Figure 5 figure supplement 1A TRF2 flag for TRF2 wt.tif]

Figure 6- A-B

Figure

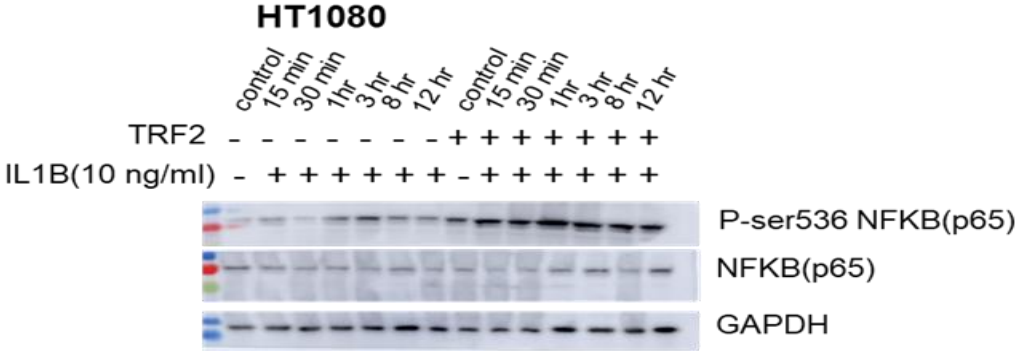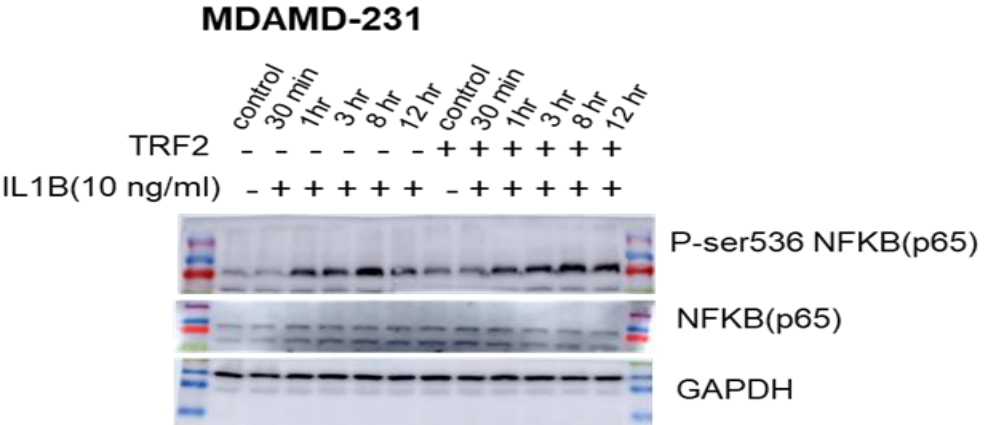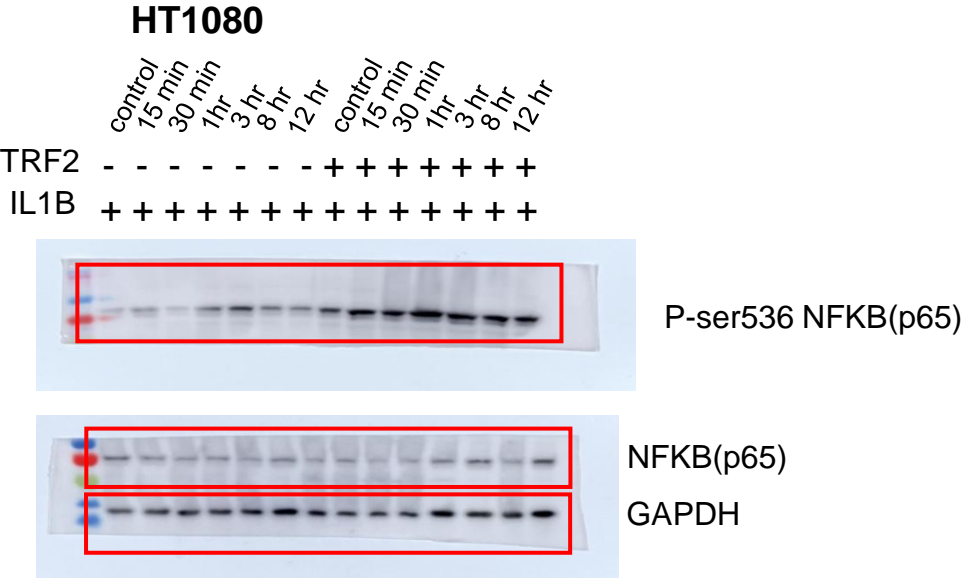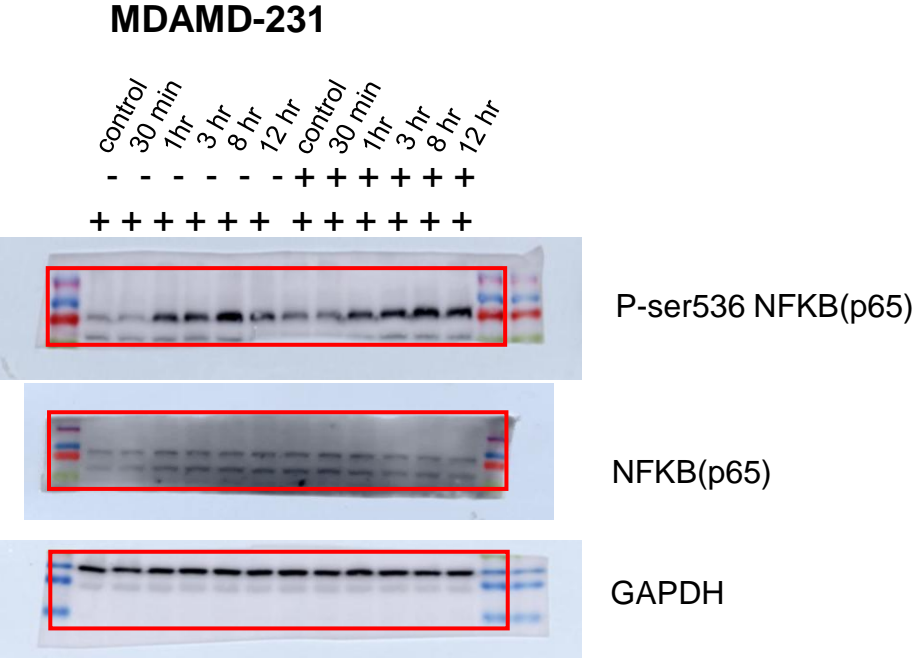

Supplement: Figure 6—source data 2. [file elife-95106-fig6-data2.zip › Figure 6__source data 2_western blots fig A_B.pdf]

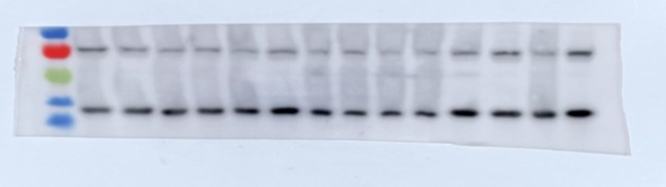

Supplement: Figure 6—source data 3. [file elife-95106-fig6-data3.zip › Figure 6_ source data 3_western blots 6 AB/Figure 6A NFKB p65 gapdh.tif]

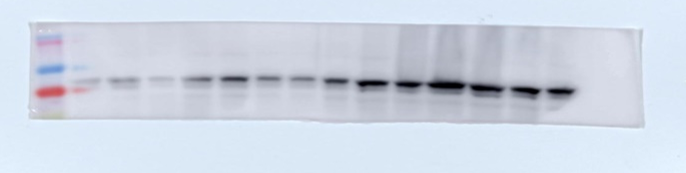

Supplement: Figure 6—source data 3. [file elife-95106-fig6-data3.zip › Figure 6_ source data 3_western blots 6 AB/Figure 6A pNFKB p65.tif]

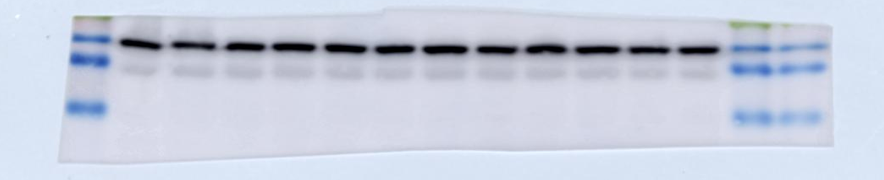

Supplement: Figure 6—source data 3. [file elife-95106-fig6-data3.zip › Figure 6_ source data 3_western blots 6 AB/Figure 6B gapdh.tif]

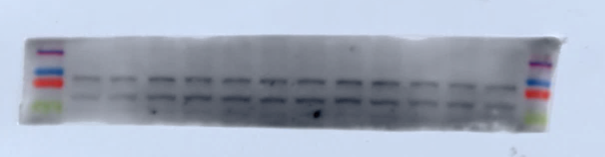

Supplement: Figure 6—source data 3. [file elife-95106-fig6-data3.zip › Figure 6_ source data 3_western blots 6 AB/Figure 6B NFKB p65.tif]

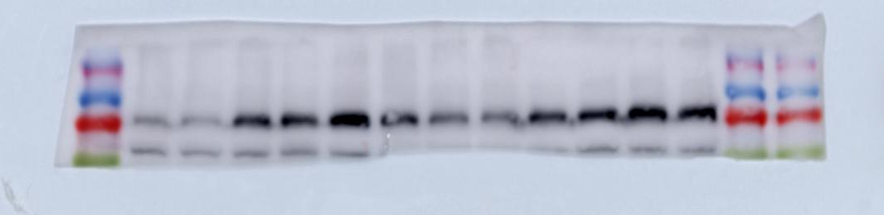

Supplement: Figure 6—source data 3. [file elife-95106-fig6-data3.zip › Figure 6_ source data 3_western blots 6 AB/Figure 6B pNFKB p65.tif]

Figure 6-figure supplement 1 B

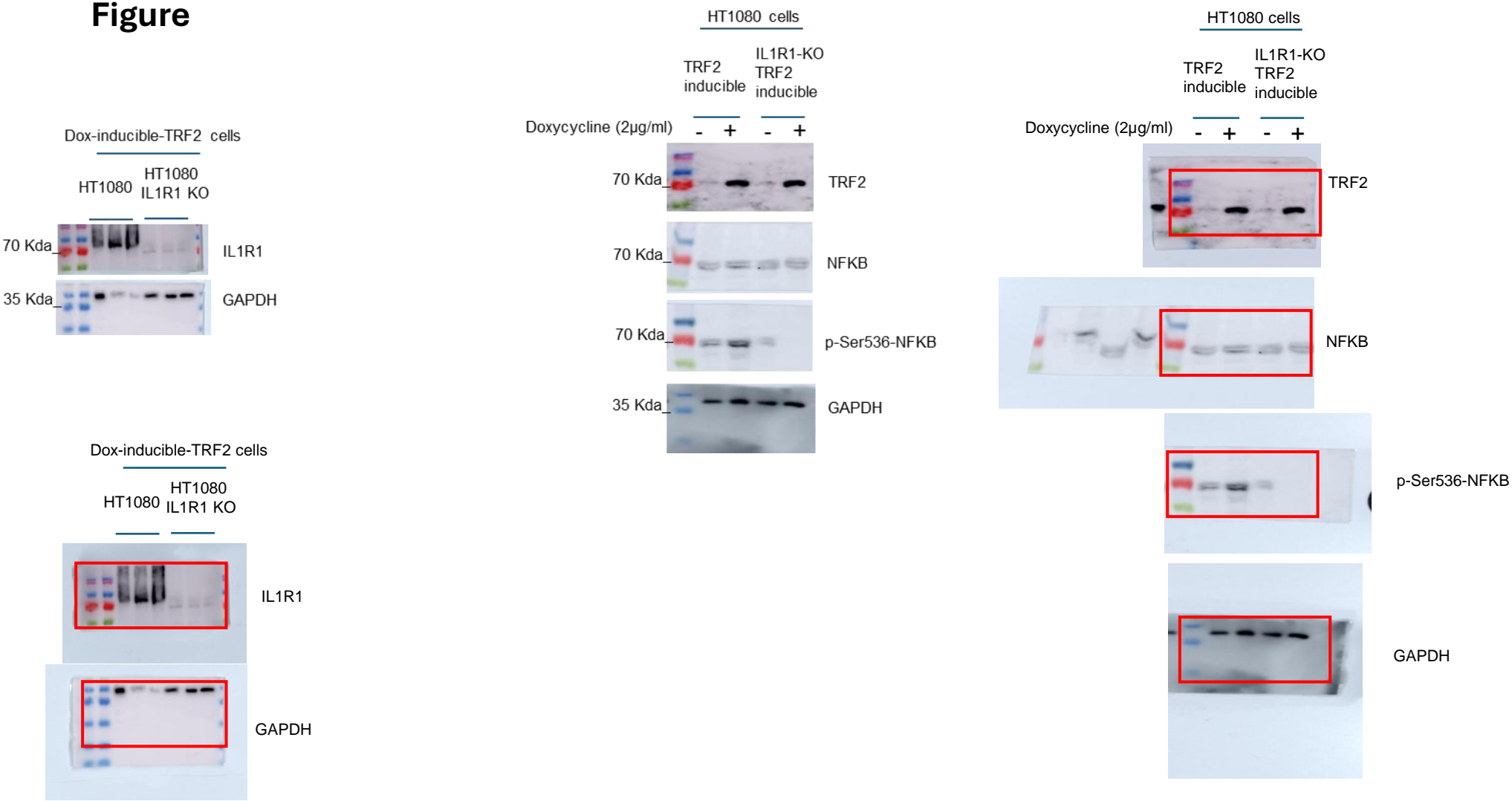

Figure 6-figure supplement 1 C

Figure

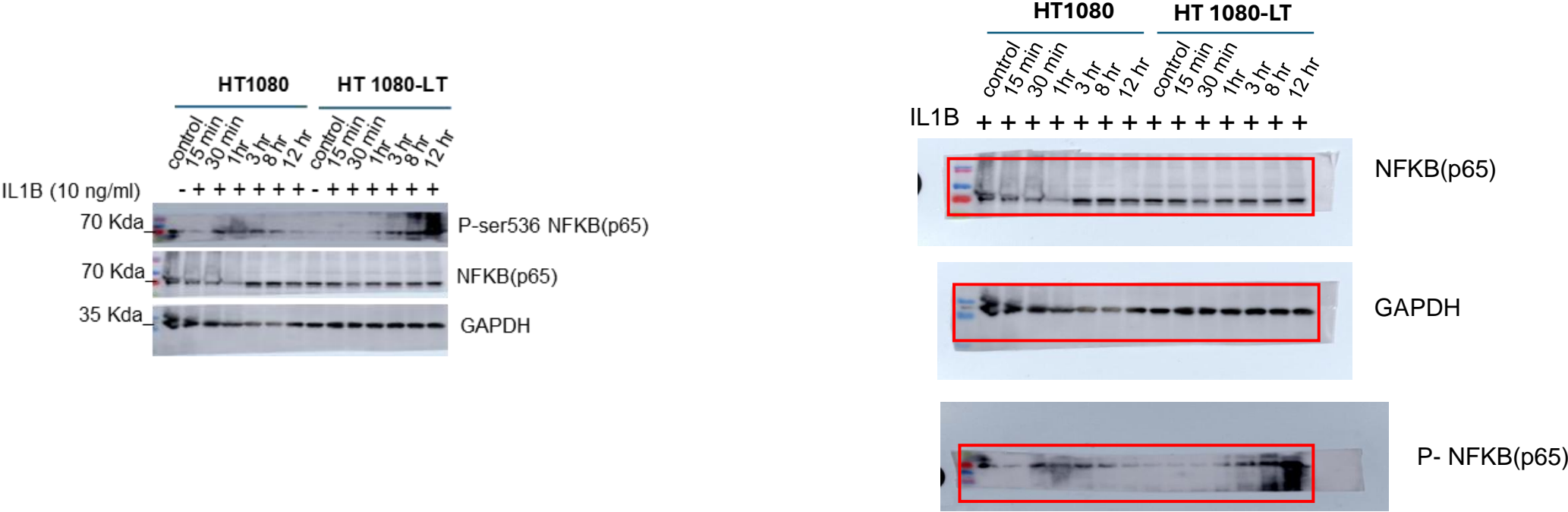

Supplement: Figure 6—figure supplement 1—source data 2. [file elife-95106-fig6-figsupp1-data2.zip › Figure 6__figure supplement 1_source data 2_western blots fig B_C.pdf]

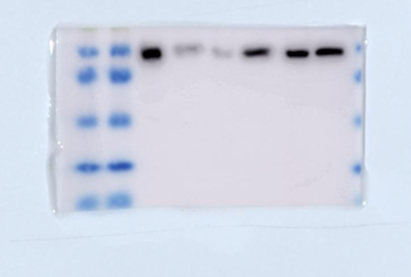

Supplement: Figure 6—figure supplement 1—source data 3. [file elife-95106-fig6-figsupp1-data3.zip › Figure 6_ figure supplement 1_source data 3_western blots fig BC/Figure 6 figure supplement 1B left GAPDH.tif]

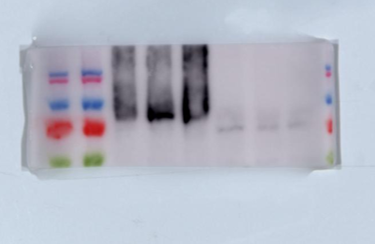

Supplement: Figure 6—figure supplement 1—source data 3. [file elife-95106-fig6-figsupp1-data3.zip › Figure 6_ figure supplement 1_source data 3_western blots fig BC/Figure 6 figure supplement 1B left IL1R1.tif]

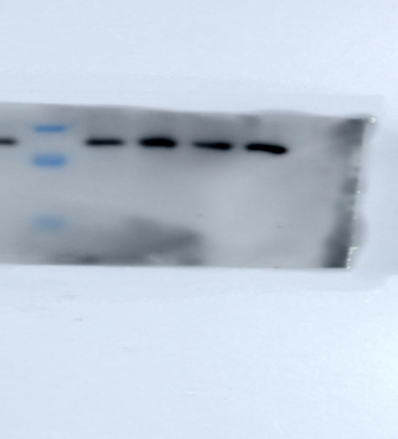

Supplement: Figure 6—figure supplement 1—source data 3. [file elife-95106-fig6-figsupp1-data3.zip › Figure 6_ figure supplement 1_source data 3_western blots fig BC/Figure 6 figure supplement 1B right GAPDH.tif]

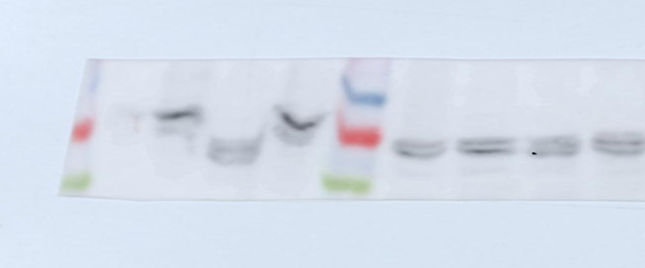

Supplement: Figure 6—figure supplement 1—source data 3. [file elife-95106-fig6-figsupp1-data3.zip › Figure 6_ figure supplement 1_source data 3_western blots fig BC/Figure 6 figure supplement 1B right NFKB P65.tif]

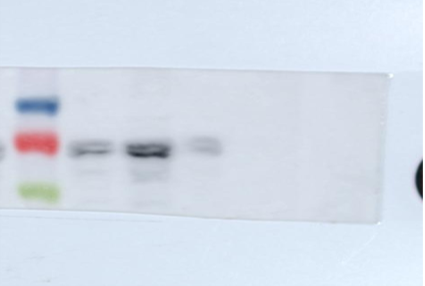

Supplement: Figure 6—figure supplement 1—source data 3. [file elife-95106-fig6-figsupp1-data3.zip › Figure 6_ figure supplement 1_source data 3_western blots fig BC/Figure 6 figure supplement 1B right PNFKB P65.tif]

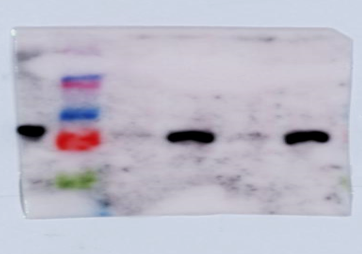

Supplement: Figure 6—figure supplement 1—source data 3. [file elife-95106-fig6-figsupp1-data3.zip › Figure 6_ figure supplement 1_source data 3_western blots fig BC/Figure 6 figure supplement 1B right TRF2.tif]

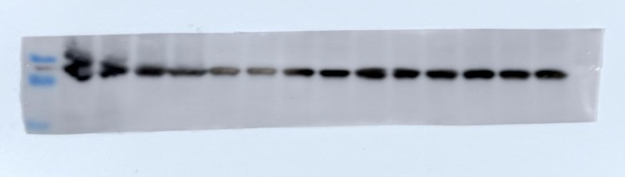

Supplement: Figure 6—figure supplement 1—source data 3. [file elife-95106-fig6-figsupp1-data3.zip › Figure 6_ figure supplement 1_source data 3_western blots fig BC/Figure 6 figure supplement 1C GAPDH.tif]

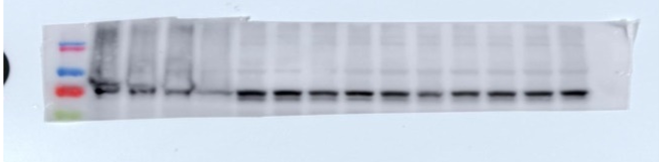

Supplement: Figure 6—figure supplement 1—source data 3. [file elife-95106-fig6-figsupp1-data3.zip › Figure 6_ figure supplement 1_source data 3_western blots fig BC/Figure 6 figure supplement 1C NFKB P65.tif]

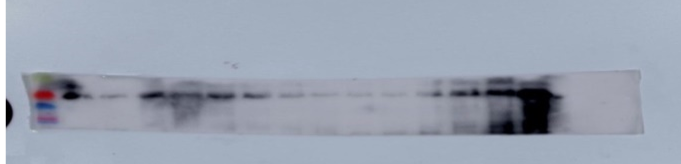

Supplement: Figure 6—figure supplement 1—source data 3. [file elife-95106-fig6-figsupp1-data3.zip › Figure 6_ figure supplement 1_source data 3_western blots fig BC/Figure 6 figure supplement 1C P NFKB P65.tif]
