# Supplementary material for: Telomere length sensitive regulation of interleukin receptor 1 type 1 (IL1R1) by the shelterin protein TRF2 modulates immune signalling in the tumour microenvironment
Source: eLife. 2024 Dec 27;13:RP95106. doi: 10.7554/eLife.95106 (PMC11677240; doi:10.7554/eLife.95106)
Supplement: MDAR checklist [file elife-95106-mdarchecklist1.docx]

**Materials Design Analysis Reporting (MDAR)**

**Materials:**

| **Newly created materials** | **Indicate where provided: section/figure legend** | **N/A** |
| --- | --- | --- |
| The manuscript includes a dedicated "materials availability statement" providing transparent disclosure about availability of newly created materials including details on how materials can be accessed and describing any restrictions on access. | Materials availability & Correspondence |  |
|  |  |  |
| **Antibodies** | **Indicate where provided: section/figure legend** | **N/A** |
| For commercial reagents, provide supplier name, catalogue number and [RRID](https://scicrunch.org/resources), if available. | Materials and methods:  Key resource table; sub-section-Antibodies |  |
|  |  |  |
| **DNA and RNA sequences** | **Indicate where provided: section/figure legend** | **N/A** |
| Short novel DNA or RNA including primers, probes: Sequences should be included or deposited in a public repository. | For primer/oligo sequences:  Materials and methods:  Sun-sections- mRNA expression primers, ChIP- qRT PCR primers,IL1R1 promoter G4 mutant HEK cells  Plasmids:  Materials and methods:  Key resource table; sub-section- plasmid vectors  raw data for the RNA sequencing (HT1080-HT1080-LT cells) has been made public (GEO accession code GSE267781). |  |
|  |  |  |
| **Cell materials** | **Indicate where provided: section/figure legend** | **N/A** |
| Cell lines: Provide species information, strain. Provide accession number in repository OR supplier name, catalog number, clone number, OR RRID. | Materials and methods:  Key resource table; sub-section- Cell lines, media and culture conditions |  |
| Primary cultures: Provide species, strain, sex of origin, genetic modification status. | Materials and methods:  Key resource table; sub-section- Organoid generation and culture |  |
|  |  |  |
| **Experimental animals** | **Indicate where provided: section/figure legend** | **N/A** |
| Laboratory animals or Model organisms: Provide species, strain, sex, age, genetic modification status. Provide accession number in repository OR supplier name, catalog number, clone number, OR RRID. | Materials and methods: sub-section-Xenograft tumour generation in NOD-SCID mice |  |
| Animal observed in or captured from the field: Provide species, sex, and age where possible. |  | NA |
|  |  |  |
| **Plants and microbes** | **Indicate where provided: section/figure legend** | **N/A** |
| Plants: provide species and strain, ecotype and cultivar where relevant, unique accession number if available, and source (including location for collected wild specimens). |  | NA |
| Microbes: provide species and strain, unique accession number if available, and source. |  | NA |
|  |  |  |
| **Human research participants** | **Indicate where provided: section/figure legend) or state if these demographics were not collected** | **N/A** |
| If collected and within the bounds of privacy constraints report on age, sex, gender and ethnicity for all study participants. | Demographic data of human subjects have not been used for this study. Relevant clearances and relevant details have been provided in the manuscript under the section - Ethics statement. |  |

**Design:**

| **Study protocol** | **Indicate where provided: section/figure legend** | **N/A** |  |
| --- | --- | --- | --- |
| If the study protocol has been pre-registered, provide DOI. For clinical trials, provide the trial registration number OR cite DOI. | Materials and methods section and Respective figure legends. |  |  |
|  |  |  |  |
| **Laboratory protocol** | **Indicate where provided: section/figure legend** | **N/A** |  |
| Provide DOI OR other citation details if detailed step-by-step protocols are available. | Materials and methods: |  |  |
|  |  |  |  |
| **Experimental study design (statistics details) *** | | |  |
| **For in vivo studies: State whether and how the following have been done** | **Indicate where provided: section/figure legend. If it could have been done, but was not, write “not done”** | **N/A** |  |
| Sample size determination | Materials and methods: sub-section-Xenograft tumour generation in NOD-SCID mice |  |  |
| Randomisation |  | NA |  |
| Blinding |  | NA |  |
| Inclusion/exclusion criteria |  | NA |  |
|  |  |  |  |
| **Sample definition and in-laboratory replication** | **Indicate where provided: section/figure legend** | **N/A** |  |
| State number of times the experiment was replicated in the laboratory. | Respective figure legends. |  |  |
| Define whether data describe technical or biological replicates. | Respective figure legends. |  |  |
|  |  |  |  |
| **Ethics** | **Indicate where provided: section/submission form** | **N/A** |  |
| Studies involving human participants: State details of authority granting ethics approval (IRB or equivalent committee(s), provide reference number for approval. | Ethics statement |  |  |
| Studies involving experimental animals: State details of authority granting ethics approval (IRB or equivalent committee(s), provide reference number for approval. | Ethics statement |  |  |
| Studies involving specimen and field samples: State if relevant permits obtained, provide details of authority approving study; if none were required, explain why. |  |  |  |
|  |  |  |  |
| **Dual Use Research of Concern (DURC)** | **Indicate where provided: section/submission form** | **N/A** |  |
| If study is subject to dual use research of concern regulations, state the authority granting approval and reference number for the regulatory approval. |  | NA |  |

**Analysis:**

| **Attrition** | **Indicate where provided: section/figure legend** | **N/A** |
| --- | --- | --- |
| Describe whether exclusion criteria were pre-established. Report if sample or data points were omitted from analysis. If yes, report if this was due to attrition or intentional exclusion and provide justification. |  | NA |
|  |  |  |
| **Statistics** | **Indicate where provided: section/figure legend** | **N/A** |
| Describe statistical tests used and justify choice of tests. | Materials and methods: sub-section -Statistical Analysis.  Respective figure legends. |  |
|  |  |  |
| **Data availability** | **Indicate where provided: section/submission form** | **N/A** |
| For newly created and reused datasets, the manuscript includes a data availability statement that provides details for access (or notes restrictions on access). | Section- Data availability |  |
| When newly created datasets are publicly available, provide accession number in repository OR DOI and licensing details where available. | Section- Data availability |  |
| If reused data is publicly available provide accession number in repository OR DOI, OR URL, OR citation. | Supplementary File 4 |  |
|  |  |  |
| **Code availability** | **Indicate where provided: section/figure legend** | **N/A** |
| For any computer code/software/mathematical algorithms essential for replicating the main findings of the study, whether newly generated or re-used, the manuscript includes a data availability statement that provides details for access or notes restrictions. |  | NA |
| Where newly generated code is publicly available, provide accession number in repository, OR DOI OR URL and licensing details where available. State any restrictions on code availability or accessibility. |  | NA |
| If reused code is publicly available provide accession number in repository OR DOI OR URL, OR citation. |  | NA |

**Reporting:**

The MDAR framework recommends adoption of discipline-specific guidelines, established and endorsed through community initiatives.

| **Adherence to community standards** | **Indicate where provided: section/figure legend** | **N/A** |
| --- | --- | --- |
| State if relevant guidelines (e.g., ICMJE, MIBBI, ARRIVE, STRANGE) have been followed, and whether a checklist (e.g., CONSORT, PRISMA, ARRIVE) is provided with the manuscript. |  | NA |
